# Novel Variants of CEP152 in a Case of Compound-Heterozygous Inheritance of Epilepsy

**DOI:** 10.1055/s-0043-1777807

**Published:** 2024-01-16

**Authors:** Weiran Li, Xiaowei Lu, Jianbo Shu, Yingzi Cai, Dong Li, Chunquan Cai

**Affiliations:** 1 Graduate College of Tianjin Medical University, Tianjin, People's Republic of China; 2 Tianjin Children's Hospital (Children's Hospital of Tianjin University), Tianjin, People's Republic of China; 3 The Medical Department of Neurology, Tianjin Children's Hospital, Tianjin, People's Republic of China; 4 Tianjin Pediatric Research Institute, Tianjin, People's Republic of China; 5 Tianjin Key Laboratory of Birth Defects for Prevention and Treatment, Tianjin, People's Republic of China; 6 Medical College of Tianjin University, Tianjin, People's Republic of China

**Keywords:** *CEP152*, compound-heterozygous, variants, phenotypes, WES, Sanger sequencing

## Abstract

**Introduction** *CEP152* encodes protein Cep152, which associates with centrosome function. The lack of Cep152 can cause centrosome duplication to fail. *CEP152* mutates, causing several diseases such as Seckel syndrome-5 and primary microencephaly-9.

**Methods** In this study, we reported a patient diagnosed with epilepsy in Tianjin Children's Hospital. We performed clinical examination and laboratory test, and whole-exome sequencing was performed for the proband's and his parents' peripheral blood. The suspected compound-heterozygous variant in the *CEP152* gene was verified by Sanger sequencing and quantitative real-time polymerase chain reaction technology.

**Results** We discovered three variants—two of them from *CEP152* and one from *HPD* . The result showed the variants in *CEP152* only. The patient presented with seizures frequently. Sanger sequencing showed two novel variants in *CEP152* are in exon26 (NM_014985.3 c.3968C > A p.Ser1323*) and in exon16 (NM_014985.3 c.2034_2036del p.Tyr678*).

**Conclusions** We reported a novel compound-heterozygous variant in the *CEP152* gene in this study. Most of the phenotypes are Seckel syndrome and primary microencephaly, and the novel variant may cause an atypical phenotype that is epilepsy.

## Introduction

The *CEP152* on chromosome 15q21 coding protein Cep152 is thought to involve centrosome function. Also, the protein Cep152 can interact with Cep57, Cep63, and Cep192 to recruit polo-like-kinase 4 (Plk4). 1 2 They (especially Cep192 and Cep152) can competitively interact with the cryptic polo box of Plk4 to form a hierarchical scaffold, while Cep152 needs Cep192 to localize at the periphery of the outer wall of centrioles. 3 4 The centrosome is the major microtubule-organizing center of animal cells, consisting of two centrioles surrounded by pericentriolar material. It has the function of division cells as it determines the poles of the mitotic spindle that segregates duplicated chromosomes between dividing cells. 5 6 7 Centrioles are found in the centrosome core, and Cep152 plays a role in open-ended cylinder-like localization pattern around centrioles. 6 8 Centrioles assembly and duplication are controlled by Plk4. Plk4 controls centrioles assemble by downregulated or overexpression, which can block and promote centrioles assemble, respectively. 3 8 Human Cep152 provides a conserved molecular platform, which acts as a scaffold for Plk4 and centrosomal P4.1-associated protein (CPAP) interaction, the amino terminus interacts with polo box of Plk4, and the carboxy terminus interacts with CPAP, a protein that controls the Plk4-regulated centriole length. 6 8 Interestingly, after Cep152 recruits Plk4 and CPAP, it can colocalize with Cep63 or centrosomal localization and interact with Cep192. Also, Cep152 requires CPAP for centrosomal loading. 2 6 8 However, Cep152 can interact with Plk4 to initiate centriole formation, and depletion of Cep152 causes centrosome duplication to fail, preventing centriole duplication and Plk4 overexpression-induced centriole amplification. 8 9

*CEP152* encodes 1654 amino acid protein, and its mutation can cause several diseases, which are Seckel syndrome-5 (OMIM 613823), primary microcephaly-9 (OMIM 614852), and so on. 10 To simplify the classical clinical phenotype, the most expected phenotypes are Seckel syndrome and primary microcephaly, as these two main kinds of phenotypes present 15 times in all 25 mutations. Seckel syndrome is an autosomal recessive disease that can cause proportionate short stature, severe microcephaly, mental retardation, and a typical “bird-head” facial appearance, which present a facial feature of the sloping forehead, high nasal bridge, beaked nose, retrognathia, and microcephaly. 10 11 12 The mutation of *Cep152* leads to more cells containing multiple nuclei and centrosomes and causes incorrect cell division, which finally leads to Seckel syndrome. 10 Microcephaly is also an autosomal recessive disease. The clinical diagnosis involves an individual exhibiting a head circumference more than three standard deviations below the mean for their age and sex, accompanied by mental retardation. This condition is identified when there are no other associated malformations or discernible etiology. 13 However, primary microcephaly is a static concept that the patient has a small but architecturally normal brain. 13 In primary microcephaly, mutant *CEP152* can cause the failure of colocalization. 14 However, other phenotypes include atrioventricular septum defect, epileptic encephalopathy, and autism spectrum disorder.

We reported a novel case of compound-heterozygous inheritance of epilepsy. Both c.3968C > A p.(Ser1323*) and c.2034_2036del p.(Tyr678*) are novel nonsense mutations. In our study, we performed whole-exome sequencing (WES) in a patient. A compound-heterozygous inheritance was identified and showed a rare phenotype that neither Seckel syndrome nor microcephaly, while a mutation has already been reported can cause Seckel syndrome. 15 A series of experiments was performed to determine the relationship between variants and phenotypes.

## Materials and Methods

### Patients and DNA Extraction

According to the manufacturer's protocol, DNA was extracted from peripheral blood samples using a blood genomic DNA Mini kit (cat. no. CW0541; CoWin Biosciences, Jiangsu, China). The ratio of the absorbance at 260 and 280 nm (A260/280 ratio) was evaluated with 1 µL of DNA extraction using the NanoDrop 2000 spectrophotometer (Thermo Fisher Scientific, Inc., Waltham, MA, USA). We obtained a total of 100 µL DNA solution (≥10 ng/µL), which was stored at −20°C.

### WES and Bioinformatics Analysis

WES for proband was performed by BGI Group. Paired-end sequencing was performed with reading lengths of 150 bp and more than 95% of the target regions, including all coding regions and exon–intron boundaries with an average coverage depth of 100-fold. Burrows-Wheeler Aligner software was used to align the sequencing data with the human reference genome hg19. Genome Analysis Toolkit software analyzed the insertion, deletion, and single nucleotide polymorphism sites. Variant annotations were made using the ANNOVAR tool (V20180118; https://doc-openbio.readthedocs.io/projects/annovar/en/latest/ ), 1,000 genomes ( https://www.1000genomes.org/ ), dbSNP ( https://www.ncbi.nlm.nih.gov/snp/?term= ), and OMIM ( https://omim.org/ ) databases. The effect of the variants on the structure and function of the proteins was predicted using polymorphism phenotyping v2 software ( http://genetics.bwh.harvard.edu/pph2/index.shtml ) and Sorts Intolerant from Tolerant software (V1.1; http://sift.jcvi.org ,).

### Variant Screening and Sanger Sequencing

Polymerase chain reaction (PCR) and further Sanger sequencing were performed to confirm the candidate variants and analyze the cosegregation pattern for both two variants, respectively. For the *CEP152* located at chromosome 15, PCR and Sanger sequencing were performed for the proband and his parents. Amplification was performed in a final volume of 50 µL, containing 25 µL 2X GC buffer I, 20 mM deoxynucleotide triphosphates mixture, 100 200 ng DNA, 0.5 µM forward and reverse primers, and 2.5 U LA Taq polymerase (cat. no. RR02AG; Takara Biotechnology Co., Ltd.). The PCR utilized an initial denaturation at 94°C for 2 minutes, followed by 35 cycles of 94°C for 30 seconds, 58°C for 30 seconds, and 72°C for 40 seconds. A final extension step at 72°C for 5 minutes concluded the thermocycling process. The PCR products were separated by 1.5% agarose gel electrophoresis. The proper DNA was purified from agarose gel using a Gel Extraction kit (CoWin Biosciences, Jiangsu, China) and sent to Genewiz (Beijing, China) for Sanger sequencing. Chromas software (version 1.62; Technelysium Pty Ltd.) was used to compare the sequencing data with the reference sequences (NM_014985.3) in GenBank ( https://www.ncbi.nlm.nih.gov/nuccore/NC_000015.10?report=genbank&from=48662534&to=48811904&strand=true )

## Results

### Clinical Description

The 5-year-old male patient's birth weight was 4.1 kg (weight-for-age z-score: 1.44), length was 50.0 cm (height-for-age z-score: 0.06), and head circumference was 34.0 cm (head circumference-for-age z-score: −0.36). The patient's weight was 19.6 kg (weight-for-age z-score: 0.43), height was 112.0 cm (height-for-age z-score: 0.33), and head circumference was 49.6 cm at the latest assessment (head circumference-for-age z-score: −0.78). 16

**Fig. 1:**
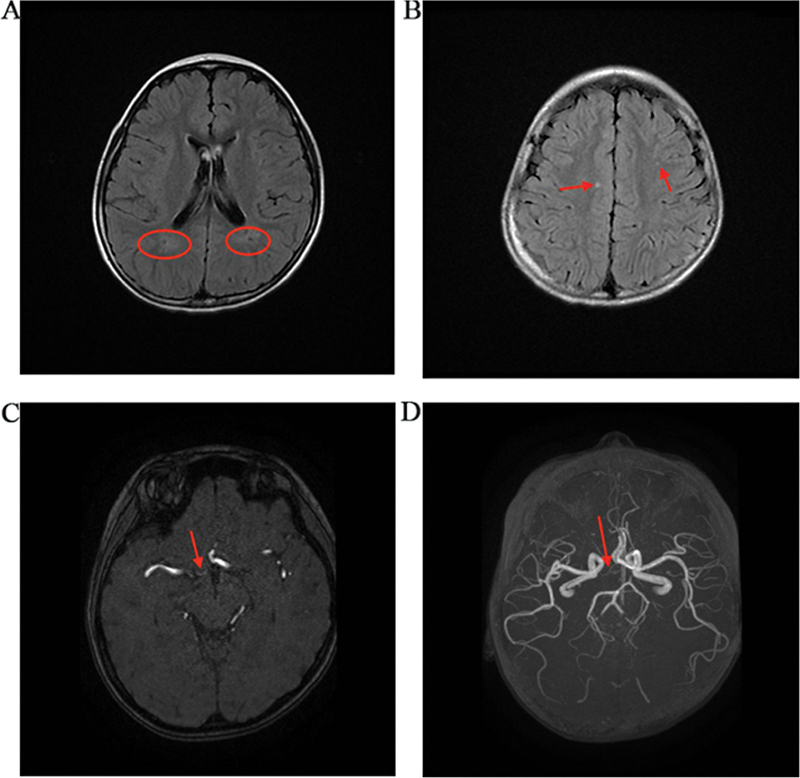
Patient's radiograph.

The patient presented to the inpatient unit with the primary complaint of recurrent seizures. The epilepsy was discovered without fever 8 months ago and happened occasionally. The epilepsy lasted a few seconds before it was eased. Four months ago, the condition progressed to both eyes staring upwards, consciousness loss, mouth corner skewed, head back without fever, and the symptoms lasted 3 to 5 minutes. Epilepsy struck the patient many times after he was admitted to the hospital. To control epilepsy, valproate sodium was used as a symptomatic therapy. After being admitted to the hospital, the symptoms progressed to both eyes looking to the right, with a regular swing to the right that lasted 2 to 5 minutes. Furthermore, vomiting occurred four times with consciousness during the last eight seizures. The vomiting continued for 30 minutes until being relieved. Physical examination revealed that the patient had a poor mental reaction, with the right eyelid lowering and the others normal. The clinical diagnosis is epilepsy because the seizures occur often. On head magnetic resonance imaging T2-weighted and fluid-attenuated inversion recovery sequence images ( Fig. 1A, B ), the main significant features are a high signal in the white matter area of the frontal–parietal lobe and the periventricular white matter area of the bilateral parietal lobe, as well as the loss of the right anterior cerebral artery on magnetic resonance angiography ( Fig. 1C, D ). As a result, the clinical diagnosis took into account the widening of the perivascular gap.

### Genetics Analysis

**Fig. 2:**
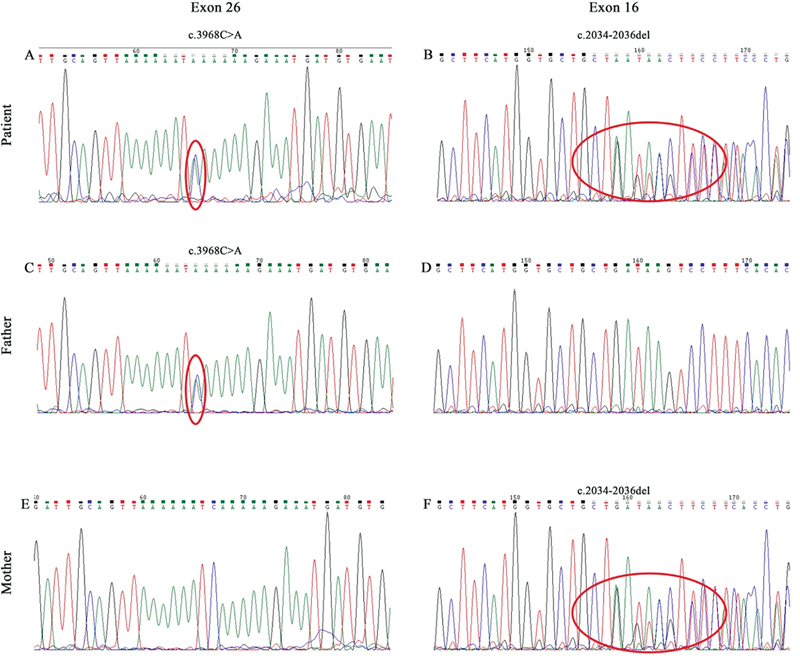
Sanger sequencing. ( **A** , **B** ) The proband's mutation. ( **C** , **D** ) The proband's father's mutations. ( **E** , **F** ) The proband's mother's mutations.

In this case, sequencing across the patient's CEP152 and HPD gene locus revealed two heterozygous variants from *CEP152* and one heterozygous variant from *HPD* . The *HPD* variant is an unclear meaning variant, so the data are not displayed. In the *CEP152* , the first variant is located in exon 26 (NM_014985.3 c.3968C > A, p.Ser1323*, Fig. 2A , ClinVar ( http://www.ncbi.nlm.nih.gov/clinvar ) Accession: SCV002499430) and the second one is in exon 16 (NM_014985.3 c.2034_2036del, p.Tyr678*, Fig. 2B , ClinVar Accession: SCV002499429). This mutation has been described previously in individuals with Seckel syndrome 15 17 18

**Fig. 3:**
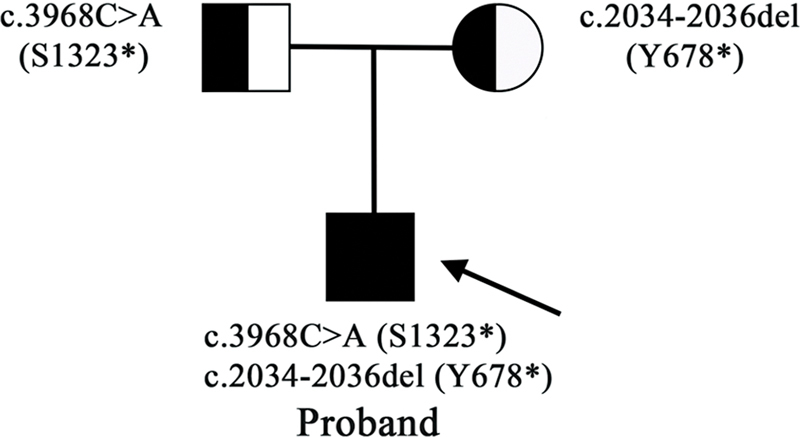
The pedigree of the proband.

The patient's father is heterozygous for the S1323* mutation ( Fig. 2C ) but does not carry the Y678* mutation ( Fig. 2D ). The patient's mother does have the S1323*mutation ( Fig. 2E ) but is heterozygous for the Y678* mutation ( Fig. 2E ). The pedigree of the compound-heterozygous patient is shown in Fig. 3 .

## Discussion

On the human gene mutation database (HGMD), 25 mutations in *CEP152* have been found. Microcephaly, both primary and congenital, and Seckel syndrome are the most common symptoms, with 15 cases reported in total. Atrioventricular septum deficiency, autistic spectrum disorder, epileptic encephalopathy, intellectual disability, and corpus callosum hypoplasia can all be caused by the other mutations.

In this case, we found the patient has a typical epilepsy symptom, and there is a case reported on HGMD that the patient has epileptic encephalopathy. Meanwhile, we considered his WES result, so we thought the atypical phenotype was acceptable. Kasak et al reported they observed a loss of function modification (c.343C > T, p.Arg115Ter), and patients showed syndromes of primary microcephaly and Seckel syndrome. The mutation can induce epileptic encephalopathy. They state that the specific phenotype is not a feature of either primary microcephaly or Seckel syndrome; in addition, the patient does not have severe microcephaly. 19 So, based on past research, epileptic encephalopathy is treated as a syndrome that can be proven.

According to the Sanger sequence, the nucleotide alterations are found at c.3968C > A and c.2034_2036del. The patient inherited one mutation from each parent, and the mutations do not reside on the same allele.

The two mutations will be discussed separately. D'Alessandro et al reported that *CEP152* could be the consequence of atrioventricular septal defect. Meanwhile, they located several mutations, but none of the mutations caused Seckel syndrome or microcephaly, which are associated with defects in *CEP152* . As a result of the c.4570A > G mutation, threonine at position 1524 changes to alanine, resulting in an atrioventricular septal defect. 17 As known, when the nonsense mutation occurs at the position of the last one exome and after the penultimate 50bps of a gene, the nonsense mutation is usually regarded as nonpathogenic because of the loss of the corresponding mRNA. But in our case, we observed the patient has an atypical syndrome whose phenotype is epileptic, with magnetic resonance displaying the loss of anterior cerebral artery A1 segment and pineal gland reveals high density at T2-weighted imaging. Regarding the previously reported, the missense mutation induces an atrioventricular septal defect. We believe that the c.3968 nonsense mutation, serine located at 1323 is pathological and the mRNA it induced will translate a pathological protein.

Interestingly, another mutation sited at c.2034_2036del, which causes the 678 ^th^ tyrosine to be replaced by termination, has been reported (rs754565020). Meanwhile, a report observed that the same position had a missense mutation, which caused the same amino acid change, then the patient showed the typical phenotype, Seckel syndrome. 15 This proteomic change is associated with Seckel syndrome. 10 So, we consider this mutation can cause Seckel syndrome because of the same amino acid change.

But in our case, the classic phenotype did not appear. According to the above findings, the compound-heterozygous causes an interesting phenomenon. These two nonsense mutations affect the phenotype collaborative; the lateral mutation causes an atypical phenotype, while the frontier mutation can induce a typical phenotype. Therefore, the brand-new or atypical phenotype, epilepsy, can be the coeffect of these two nonsense mutations. Also, we assume epilepsy can be the phenotype of some function area insufficiency or loss. The compound heterozygote has a feature that is the dose–effect relationship. Although the c.2034_2036del nonsense mutation induces the Seckel syndrome, the c.3968C > A nonsense mutation can terminate transcription earlier than the mRNA degradation. Therefore, depending on mRNA degradation, the typical phenotype does not appear; instead, the frequent happening of epilepsy. Within all the symptoms, we noticed that epilepsy often occurred on the right side, such as head swing to the right, which may explain the loss of the right anterior cerebral artery.

We also consider the probability that the patient may not exhibit the clinical feature, suggesting that degradation occurs before mRNA can express its function. But we still need further research to prove our hypothesis.

Overall, the patient reveals a series of new phenotypes because of the variants of *CEP152* . The atypic phenotype, which is epilepsy, can reflect *CEP152* causing nervous system development disorder. In this compound-heterozygous patient, the mechanism needs further research.
